# Homeschooling during the SARS-CoV-2 pandemic: the role of students’ trait self-regulation and task attributes of daily learning tasks for students’ daily self-regulation

**DOI:** 10.1007/s11618-021-01011-w

**Published:** 2021-04-01

**Authors:** Friederike Blume, Andrea Schmidt, Andrea C. Kramer, Florian Schmiedek, Andreas B. Neubauer

**Affiliations:** 1 DIPF | Leibniz-Institut für Bildungsforschung und Bildungsinformation, Rostocker Str. 6, 60323 Frankfurt/Main, Germany; 2 Center for Research on Individual Development and Adaptive Education of Children at Risk (IDeA), Rostocker Str. 6, 60323 Frankfurt/Main, Germany; 3 Goethe-University, Theodor-W.-Adorno-Platz 1, 60323 Frankfurt/Main, Germany

**Keywords:** Ambulatory assessment, Homeschooling, SARS-CoV‑2 pandemic, Self-regulation, Task attributes, Ambulatorisches Assessment, Homeschooling, SARS-CoV-2-Pandemie, Selbstregulation, Aufgabeneigenschaften

## Abstract

As a means to counter the SARS-CoV‑2 pandemic, schools were closed throughout Germany between mid-March and end of April 2020. Schooling was translocated to the students’ homes where students were supposed to work on learning tasks provided by their teachers. Students’ self-regulation and attributes of the learning tasks may be assumed to have played important roles when adapting to this novel schooling situation. They may be predicted to have influenced students’ daily self-regulation and hence the independence with which they worked on learning tasks. The present work investigated the role of students’ trait self-regulation as well as task difficulty and task enjoyment for students’ daily independence from their parents in learning during the homeschooling period. Data on children’s trait self-regulation were obtained through a baseline questionnaire filled in by the parents of 535 children (*M*_*age*_ = 9.69, *SD*_*age*_ = 2.80). Parents additionally reported about the daily task difficulty, task enjoyment, and students’ learning independence through 21 consecutive daily online questionnaires. The results showed students’ trait self-regulation to be positively associated with their daily learning independence. Additionally, students’ daily learning independence was shown to be negatively associated with task difficulty and positively with task enjoyment. The findings are discussed with regard to students’ daily self-regulation during the homeschooling period. Finally, implications for teaching practice during the pandemic-related school closures are derived.

## Abstract (de)

Um die SARS-CoV-2-Pandemie einzudämmen, wurden zwischen Mitte März und Ende April 2020 deutschlandweit alle Schulen geschlossen. Während dieser Zeit wurden die Schüler*innen zu Hause beschult (‚Homeschooling‘) und arbeiteten an Lernaufgaben, die von ihren Lehrkräften bereitgestellt wurden. Bei der Anpassung an diese neuartige Beschulungssituation könnten sowohl die Selbstregulation der Schüler*innen als auch die Eigenschaften der Lernaufgaben eine wichtige Rolle gespielt haben. Es ist anzunehmen, dass diese insbesondere die tagtägliche Selbstregulation der Schüler*innen beim Bearbeiten der Lernaufgaben und damit die Selbstständigkeit, mit der sie an den Lernaufgaben arbeiteten beeinflusst haben. Die vorliegende Arbeit untersuchte, welche Rolle die dispositionelle Selbstregulation von Schüler*innen, die Schwierigkeit der Lernaufgaben und die Freude an den Aufgaben für die tagtägliche Selbstständigkeit der Bearbeitung der Aufgaben während des Homeschoolings spielten. Die dispositionelle Selbstregulation der Schüler*innen wurde zunächst über einen Eingangsfragebogen erfasst, der von den Eltern von 535 Kindern ausgefüllt wurde (*M*_*Alter*_ = 9,69; *SD*_*Alter*_ = 2,80). Die Eltern berichteten anschließend an 21 aufeinander folgenden Tagen über die tägliche Aufgabenschwierigkeit, die Freude an den Aufgaben und die Selbstständigkeit, mit der ihre Kinder die Lernaufgaben bearbeiteten. Die Ergebnisse zeigten, dass die dispositionelle Selbstregulation der Schüler*innen positiv mit ihrer täglichen Selbstständigkeit beim Lernen assoziiert war. Darüber hinaus war die tägliche Selbstständigkeit der Schüler*innen beim Lernen negativ mit der Aufgabenschwierigkeit und positiv mit der Freude an den Aufgaben verbunden. Die Ergebnisse werden im Hinblick auf die tägliche Selbstregulation der Schüler*innen während der Beschulung zu Hause diskutiert. Abschließend werden Implikationen für die Lehrpraxis während der pandemiebedingten Schulschließungen abgeleitet.

## Introduction

When the SARS-CoV‑2 pandemic reached Germany at the beginning of 2020 and the number of infections increased sharply during the first wave of infections, far-reaching measures aiming at severely restricting contact between people were taken to contain the virus. As a particularly invasive measure, schools throughout Germany were closed completely between mid-March and end of April 2020. Schooling was translocated to the students’ homes and teachers provided learning materials to be worked on (Huber et al. 2020). Some teachers additionally offered project works and online lessons. However, as the homeschooling situation was entirely novel for all students and came at them largely unprepared, many of them experienced it as a major challenge. Students reported to have struggled with structuring their day, initiating their daily learning sessions, and focusing on the school work, for instance (Huber et al. 2020). Students’ self-regulation may be assumed to have played an important role while adapting to this novel schooling situation. The present work therefore investigated the role of both personal characteristics and features of school tasks students worked on during the SARS-CoV‑2 induced homeschooling period in Germany in the beginning of 2020 for students’ daily self-regulation during learning.

### Students’ trait self-regulation and academic outcomes

Some individuals can easily focus their attention, manage their emotions, and induce behaviours that support moving towards personal goals. However, others have difficulties concentrating, managing emotional reactions, and behaving appropriately, which results in difficulties in pursuing their goals. This is to say, individuals differ in their capacity to self-regulate (Duckworth et al. 2014; Gross 2015; McClelland et al. 2018). Self-regulation refers to the dynamic process of defining a goal and subsequently adjusting cognitions, emotions, and behaviour to move towards this goal (Baumeister et al. 1994; Carver and Scheier 1998; Gross 2015). It thereby encompasses a wide range of measures to be taken by an individual in pursuing an objective. Self-regulation includes selecting and setting goals, monitoring progress, and taking action to move towards goals, as well as shielding goals from competing matters (e.g., Fujita 2011; Gollwitzer 1999; Shah et al. 2002). As such, self-regulation is relevant in a vast number of situations, including such that require conflict resolution between immediately gratifying and enduringly valued goals (i.e., this subdomain of self-regulation is often termed self-control; Baumeister et al. 1994; Duckworth et al. 2014; Inzlicht et al. 2020). Self-regulation is thereby particularly important in learning situations, in which it is assumed to support students’ independent pursuit and attainment of learning goals (this subdomain of self-regulation is related to the concept of self-regulated learning; e.g., Corno and Mandinach 2004; Duckworth et al. 2014; Pintrich 2000; Schunk and Zimmerman 1994). The term *self-regulation* as used in the present article consequently denotes an individual’s disposition to orchestrate cognitions, emotions, and behaviour such that they support the pursuit of goals in diverse conflictual and conflict-free situations, including learning situations. Self-regulation may therefore be assumed to have played an important role for students’ adjustment to homeschooling during the first wave of infections of the SARS-CoV‑2 pandemic, supporting the initiation of daily learning sessions and the pursuit of learning goals, for instance.

The significance of students’ trait self-regulation for their academic success has consistently been demonstrated (Dent and Koenka 2016; Duckworth et al. 2019; Willoughby et al. 2019). Considering longer time frames and longitudinal data, students with higher trait self-regulation have been shown to achieve better grades and to obtain higher educational attainment overall (de Ridder et al. 2012; Mischel et al. 1988; Moffitt et al. 2011; Shores and Shannon 2007; Wolfe and Johnson 1995). In contrast, self-regulation difficulties have been demonstrated to increase the probability of repeating a grade and being diagnosed with special education needs (Daley and Birchwood 2010; Polderman et al. 2010). Research considering learning situations spanning shorter time frames (e.g., single lessons, learning episodes) and learning contexts inside and outside the classroom (e.g., during homework, in distance education settings) further supported the relevance of students’ trait self-regulation for their learning outcomes (e.g., Blume et al. 2019; Dabbagh and Kitsantas 2004; Trautwein and Köller 2003).

In the research context, self-regulation has often been conceptualised as a disposition that is relatively stable across time and context and hence hardly malleable in the short term (e.g., Duckworth et al. 2014; Moffitt et al. 2011; Pintrich and De Groot 1990; Tangney et al. 2004). As such, self-regulation was usually assessed at only one point in time, which does not give justice to potential fluctuations within individuals, however. Nevertheless, it is well established today that self-regulation also varies within individuals, hence fluctuating over time (Liborius et al. 2019; Ludwig et al. 2016; Schmid et al. 2020; Schmitz and Wiese 2006). Empirical evidence furthermore suggested that individual momentary self-regulation varies in response to characteristics of the situation currently experienced. In the school context, students’ self-regulation was shown to differ during small group and individual work as well as while working on easier and more difficult tasks, for instance (Horvath et al. 2006; Imeraj et al. 2013). Additionally, students’ self-regulation was shown to be responsive to whether learning goals were pursued out of interest and enjoyment (i.e., autonomous goals) or rather for external reasons (e.g., to please others, obtain external reward; Judge et al. 2005; Koestner et al. 2008; Milyavskaya et al. 2015; Muraven et al. 2008; Sieber et al. 2019). Consequently, self-regulation should be understood as to encompass both a trait and a state component. Single-assessment trait self-regulation measures might therefore be associated with measures capturing individual momentary self-regulation, for instance in learning situations. However, whether single assessments of trait self-regulation and aggregated repeated assessments of state self-regulation in learning situations actually converge has only rarely been investigated so far (Martin et al. 2015; Murayama et al. 2017). An association between trait and repeated state assessments could nevertheless explain why trait self-regulation was shown to be related to individual learning success over shorter periods of time (e.g., Blume et al. 2019; Dabbagh and Kitsantas 2004; Trautwein and Köller 2003). The present work therefore aimed to examine the association between a single assessment measure of trait self-regulation and aggregated values of an indicator of daily self-regulation during the SARS-CoV‑2 induced homeschooling period.

### Situational and instructional effects on students’ state self-regulation

In view of the presumed relevance of students’ daily self-regulation for their academic success, teachers should aim to support this capacity in learning situations as best as possible. Given the assumption that self-regulation is malleable through attributes of the learning context, teachers’ systematic management and design of the learning environment should be one way to achieve this goal. Indeed, aspects of instructional quality and the ways it can be achieved are largely well established today, while there is, nevertheless, a plethora of models differing in conceptualisations and hence recommendations on how high-quality teaching can be achieved (Charalambous and Praetorius 2018; Praetorius et al. 2018). Adopting a three-dimensional model of instructional quality with classroom management, instructional, and emotional support as its integral parts (alternative terms: classroom management, cognitive activation, constructive support; Kunter and Trautwein 2013; La Paro et al. 2004; Seidel and Shavelson 2007), first studies suggested high instructional quality to be positively associated with students’ self-regulation (Rieser et al. 2013; Rimm-Kaufman et al. 2009). In particular, teachers’ effective classroom management was shown to benefit students’ behavioural self-regulation, while both instructional and emotional support were shown to benefit their cognitive self-regulation. These results are in accordance with what has also been demonstrated for students with self-regulation difficulties (Brody et al. 2002; Dolezal et al. 2003; McWilliam et al. 2003; Zentall 2005).

However, the nationwide school closures from mid-March to end of April 2020 in Germany translocated schooling to students’ homes. This entirely novel situation left teachers and school boards with only few answers to how students’ self-regulation and thus learning could best be supported. During the school closures, teachers provided their students with learning materials either on a daily or weekly basis (Huber et al. 2020). Some teachers additionally offered online lessons and project work. Nevertheless, students were supposed to work on the learning materials largely independently, with the possibility of being helped by their parents. One may assume that parental support was particularly required when students experienced self-regulation difficulties while working on the daily learning tasks. Self-regulation difficulties during learning should be expected to lead to difficulties with, for instance, focusing on and engaging with the tasks, as well as with persisting in the face of difficulties, and hence with progressing towards the learning goal (e.g., Corno and Mandinach 2004; Duckworth et al. 2019; Schunk and Zimmerman 2012). As a consequence, students would ask their parents for help, which could be provided in the form of one-to-one interactions about the learning content, scaffolding, feedback, or encouragement, for instance (Allen et al. 2013; Grolnick and Slowiaczek 1994; Pianta et al. 2008; J. Xu and Corno 1998). Empirical findings supported this expectation, demonstrating that students with lower self-regulation during learning asked for assistance more often, while, however, being less able to precisely voice their needs, which should additionally reduce the effectiveness of the support they receive (DuPaul and Stoner 2014; Karabenick and Gonida 2018; Marchand and Skinner 2007; Newman 2008). While this new learning situation might have been challenging for all students, it might thus have particularly been for those with self-regulation difficulties (Huber et al. 2020).

Previous research suggested perceived task difficulty to be among the decisive factors influencing students’ momentary self-regulation during learning, and thus parental support required, hence denoting a presumably important feature of the learning materials provided during homeschooling (Horvath et al. 2006; Ramdass and Zimmerman 2011; Zentall 2005). Boekaerts and Corno (2005) suggested learners to evaluate the perceived task difficulty in relation to their own abilities. Under appropriate conditions of task difficulty, self-regulation should be facilitated and students would easily engage with the learning task, thus effectively pursuing their learning goals and limiting the need for parental support (Karabenick and Gonida 2018; Newman 2008). However, when tasks are perceived to be either too easy or too difficult, students should experience difficulties with engaging with the task, hence indicating impaired state self-regulation (Boekaerts 1993; Boekaerts and Corno 2005; Zentall 2005). First, too easy tasks may be assumed to lack stimulation, whereby they might be perceived as boring or monotonous (Moneta and Csikszentmihalyi 1996; Reid 1986; Vodanovich 2003), which was shown to reduce students’ self-regulation (Pekrun 2006; Wolters 2003). Additionally, too easy tasks might implicitly signal students that it is not necessary to pay close attention, concentrate on the instruction, or actively engage with the learning material in order to successfully work on it, thus discouraging students from self-regulating (Locke and Latham 1990). In contrast, too difficult tasks may be expected to reduce students’ state self-regulation when they realise that even when trying very hard, they will not efficiently move towards their learning goals (Locke and Latham 1990). As a consequence, the probability of disengaging from the task at hand to follow immediately more gratifying matters should increase, indicating reduced state self-regulation (Boekaerts and Corno 2005). The relation between task difficulty and students’ actual self-regulation and thus independence with which the learning tasks are worked on is therefore expected to be curvilinear. In particular, self-regulation and thus the degree of learning independence should peak around optimal task difficulty levels and decrease when difficulty deviates in either direction (Fernyhough and Fradley 2005; van Steenbergen et al. 2015; Wodka et al. 2009). Additionally, as the fit between students’ abilities and task difficulty might be expected to be even more important for students with generally lower trait self-regulation, one may expect their state self-regulation to decline even more with less adequate fit than that of students with higher trait self-regulation (Zentall 2005). Under such circumstances, students will increasingly turn to their parents for assistance (Grolnick and Slowiaczek 1994; Karabenick and Gonida 2018; Newman 2008; J. Xu and Corno 1998). Consequently, students’ trait self-regulation may be expected to moderate the curvilinear association of task difficulty and students’ state self-regulation.

As a further feature of the learning material, task enjoyment was suggested to be relevant for students’ momentary self-regulation during learning (Judge et al. 2005; Koestner et al. 2008). Studies investigating the relevance of the quality of goal motivation for the probability of goal attainment demonstrated that goals pursued out of interest and enjoyment (i.e., autonomous goals, ‘want-to’ goals) increased momentary self-regulation (Milyavskaya et al. 2015; Muraven et al. 2008; Sieber et al. 2019). They were thus more likely to be attained as compared to goals pursued for external reasons (e.g., to please others, obtain external reward; i.e., controlled goals, ‘have-to’ goals). Consequently, more enjoyable learning tasks should be expected to support students’ momentary self-regulation and thus independence with which they worked on learning tasks. Theoretical considerations suggested that task enjoyment might even (partially) compensate for deficient state self-regulation resulting from inadequate task difficulty (Baumeister and Vohs 2007). Whether higher task enjoyment could, however, compensate for negative effects of inadequate task difficulty on students’ daily self-regulation during homeschooling, is furthermore yet to be determined.

### The present study

The present study aimed to investigate the relevance of trait self-regulation and perceived task difficulty for students’ daily self-regulation during homeschooling during the SARS-CoV‑2 pandemic between end of March and end of April 2020. Students’ daily self-regulation during learning was thereby assessed as the independence from their parents and absence of problems with which they worked on the learning tasks. Additionally, it examined the role of task difficulty and task enjoyment for students’ learning independence.

In particular, we (a) hypothesized a positive association between students’ *trait self-regulation* and the *daily learning independence *during homeschooling. Additionally, we (b) anticipated a negative quadratic association of the perceived* task difficulty* of the learning tasks with students’ *daily learning independence*, indicating that daily learning independence would be lower in situations of both too easy and too difficult learning material. We further (c) expected this quadratic association to be more pronounced in students with lower *trait self-regulation*. Finally, we (d) hypothesized the relevance of *task enjoyment* experienced during daily schoolwork as a moderator of the relation between perceived* task difficulty* of the learning tasks and students’ *daily learning independence*, with a weaker association between perceived task difficulty of the learning tasks and students’ learning independence on days students experienced more task enjoyment.

## Methods

The data analysed in the present study were collected as part of a project aiming to examine the adjustment of parents of schoolchildren to the measures taken to counter the SARS-CoV‑2 pandemic (PACO: Psychological Adjustment to the COVID-19 pandemic). Only procedures and measures relevant for the present investigation will be reported in detail here. More detailed information on the study protocol and all variables obtained can be viewed from the Open Science Framework (OSF) repository, https://osf.io/86upz. All data and the analysis code required to reproduce the results can be found there as well. The study was approved by the local ethics committee.

### Sample

Parents of schoolchildren were recruited via announcements posted to social media platforms, contacts to schools and parent-teacher associations, as well as a press release issued by the authors’ institution, which was further distributed through German newspapers. A total of 970 parents participated in an online baseline questionnaire. However, for the present analyses only data of parents who additionally participated in the subsequent daily diary phase, spanning 21 days, and who completed at least one daily assessment during which they informed about their youngest schoolchild were included. This yielded a sample of 535 participants. Participating parents were predominantly female (87.9%) and on average 42.78 years old (*SD*_*age*_ = 6.09, range = 25–63). Most parents had a university degree (*n* = 330; 61.7%), followed by an advanced school degree (*n* = 88; 16.4%), or an intermediate secondary school degree (*n* = 67; 12.5%). Fifty participants (9.3%) reported other highest attained school degrees. Net monthly household income was reported as 4000 € or higher by 264 (49.3%) of the participants; 75 participants (14.0%) reported incomes less than 2500 € and 138 (25.8%) between 2500 and 4000 € (no information on income was reported by 38 participants, 7.1%). Data on the youngest schoolchild living in the participants’ household was collected. Most of the schoolchildren included in the analyses (256 female, 47.85%; *M*_*age*_ = 9.69, *SD*_*age*_ = 2.80, range = 6–19) attended elementary school (*n* = 356, 66.5%), 127 children attended the academic tier of secondary school (‘Gymnasium’; 23.7%), and 52 children (9.7%) a different school form in the current school year.

### Procedure

Parents interested in participating in the study accessed the baseline questionnaire, which was provided through SoSci Survey (Leiner 2019), between 27 March and 3 April 2020. They were eligible to fill in the questionnaire when being 18 years or older and living in a household with a schoolchild. Only one member of a household could participate. Following informed consent, parents reported about background variables concerning themselves, the family, and their youngest schoolchild (e.g., age, school form, and grade attended), as well as his or her trait self-regulation. Additional constructs assessed can be seen in the study protocol provided on OSF, https://osf.io/86upz. Subsequently, parents could register for a second part of the study, including a daily diary study over the following 21 days and a post-assessment on day 22. Upon registration, they received a link to an online questionnaire every evening at 7 p.m., which they could access until 5 a.m. the next morning. They were instructed to fill in the questionnaire before going to bed. Completing the questionnaire took approximately 5–7 min. To remunerate participation in the baseline interview, parents could take part in a lottery, where 40 retail vouchers with a value of 50 € each were raffled. For the second study part, all parents could take part in a lottery for three iPads and another 100 retail vouchers with a value of 50 € each. With each completed daily diary, they were reimbursed with one ticket for the lottery. Completion of the post-assessment was reimbursed with two tickets. Of the potentially possible 11,235 daily questionnaires (535 * 21), 7581 were filled in, corresponding to an overall compliance rate of 67.5%.

### Measures

All child-related data obtained refer to the youngest schoolchild in the household of the participating parents.

#### Baseline measure

Students’ trait self-regulation was assessed using the hyperactivity subscale of the German version of the Strength and Difficulties Questionnaire (SDQ-Deu), which also includes items on inattention and impulsivity (Goodman 1997; Woerner et al. 2002). Parents were asked to rate to what extent five statements (i.e., ‘Restless, overactive, cannot sit still for long’, ‘Constantly fidgeting or squirming’, ‘Easily distracted, concentration wanders’, ‘Thinks things out before acting’, ‘Sees tasks through to the end, good attention span’) were true for their child (0 = not true; 1 = somewhat true; 2 = completely true), thereby relating to the last week. Responses to the first three items were recoded so that higher values indicated greater self-regulation abilities (note the difference to the standard evaluation, where the last two items would be recoded so that higher values indicated larger self-regulation difficulties). Answers were then averaged across the five items and multiplied by five. Scores could thereby range between 0 and 10, with higher scores indicating better self-regulation. Internal consistency of the scale in the analytic sample reported here was estimated as McDonald’s ω = 0.83.

#### Daily measures

During the daily diary phase, parents were asked each day whether their child had worked on materials or tasks for school that day^1^. Only when parents indicated their children had worked on materials or tasks for school that day were additional items about the learning material queried, and only those days were considered in all present analyses (*n* = 3092 observations).

##### Daily learning independence

Students’ daily independence from their parents in learning was assessed with two items asking about the degree to which they (a) had worked independently on the learning tasks or (b) had required help from their parents that day. Parents were asked to rate to what extent these two statements were true for their child (1 = completely disagree; 7 = completely agree). Responses to the second item were recoded. The two items were strongly correlated both on the within-person level, *r* = 0.70, and on the between-person level, *r* = 0.93, and answers were averaged. Scores could thereby range between 1 and 7, with higher scores indicating greater learning independence.

##### Task difficulty

Students’ perception of the task difficulty of the learning tasks they worked on that day was assessed using one item. Parents were asked to rate the task difficulty perceived by the child on a scale ranging from ‘too easy’ (coded as −3) to ‘too difficult’ (coded as +3). Negative scores indicated that tasks were too easy, positive scores indicated they were too difficult, and a score of 0 indicated that children perceived task difficulty to be just right.

##### Task enjoyment

Students’ task enjoyment of the learning tasks they worked on that day were assessed using one item. Parents were asked to rate to what extent the statement ‘My child enjoyed working on the learning materials/tasks today’ was true (1 = completely disagree; 7 = completely agree).

##### Covariates

Students’ age, gender, school type attended, and parental education were considered as covariates in the analyses and models presented below. Task volume was included as an additional covariate. Task volume was assessed in the daily diary part along task difficulty and was rated on a scale ranging from ‘too little’ (coded as −3) to ‘too much’ (coded as +3), with a score of 0 representing adequate task volume.

### Data analysis

Multilevel models with daily observations (Level 1) nested in participants (Level 2) were used to account for the dependency of repeated observations. To approach the first research question, a multilevel model with student *j*’s learning independence on day *d* as the dependent variable ($LI_{dj}$) and student *j*’s trait self-regulation ($\text{traitSR}_{j}$; centred on the grand mean) as predictor was set up. In addition, each students’ gender ($\text{gender}_{j}$; coded as 0 for male and 1 for female) and age ($\mathrm{age}_{j}$; centred on the grand mean) were added as covariates, as was the type of school each child attended ($\text{schooltype}_{j}$, dichotomized; coded 0 for the academic tier of secondary school, 1 otherwise) and the highest degree obtained by the reporting parent ($\text{parentdegree}_{j}$, dichotomized; coded 0 if the highest obtained degree was a university degree, 1 otherwise).

For the second set of analyses, additional predictors were entered into the model. First, a linear and a quadratic effect of task difficulty ($\mathrm{Diff}.\mathrm{lin}_{dj}$ and $\mathrm{Diff}.sq_{dj}$) and task volume ($\mathrm{Vol}.\mathrm{lin}_{dj}$ and $\mathrm{Vol}.sq_{dj}$) assessed on the same day (Model 1) were added. Next, the interaction of trait self-regulation with both the linear and the quadratic effect of task difficulty was added to test the hypothesis that the association between task difficulty and learning independence was more pronounced in children with lower trait self-regulation (Model 2). In the next model (Model 3), trait self-regulation (and the interaction with trait self-regulation) was removed and task enjoyment was added. Specifically, we added task enjoyment both as a time-varying predictor (i.e., student *j*’s task enjoyment on day *d*; $\mathrm{joy}_{dj}$) and as a person-level predictor (student *j*’s average task enjoyment across all days; $\mathrm{joy}.\text{pmean}_{j}$). The time-varying predictor was centred on the person-mean, and the person-level predictor was centred on the grand mean to allow for an interpretation of these effects as pure within-person and between-person effects, respectively (Wang and Maxwell 2015). Interactions of both predictors with both the linear and quadratic effects of task difficulty were added as well. In a final model (Model 4), we combined Models 2 and 3, that is, both trait self-regulation and task enjoyment (main effects and interactions with linear and quadratic effects of task difficulty) were entered simultaneously. The formal description of Model 4 is as follows:

Level 1:

1 $$LI_{dj}=\beta _{0j}+\beta _{1j}\cdot \mathrm{Vol}.\mathrm{lin}_{dj}+\beta _{2j}\cdot \mathrm{Vol}.sq_{dj}+\beta _{3j}\cdot \mathrm{Diff}.\mathrm{lin}_{dj}+\beta _{4j}\cdot \mathrm{Diff}.sq_{dj}+\beta _{5j}\cdot \mathrm{joy}_{dj}+\beta _{6j}\cdot \left(\mathrm{Diff}.\mathrm{lin}_{dj}x\mathrm{joy}_{dj}\right)+\beta _{7j}\cdot \left(\mathrm{Diff}.sq_{dj}x\mathrm{joy}_{dj}\right)+\varepsilon _{dj}$$

Level 2:

2 $$\beta _{0j}=\gamma _{00}+\gamma _{01}\cdot \mathrm{age}_{j}+\gamma _{02}\cdot \text{gender}_{j}+\gamma _{03}\cdot \text{schooltype}_{j}+\gamma _{04}\cdot \text{parentdegree}_{j}+\gamma _{05}\cdot \mathrm{joy}.\text{pmean}_{j}+\gamma _{06}\cdot \text{traitSR}_{j}+\upsilon _{0j}$$

3 $$\beta _{1j}=\gamma _{10}$$

4 $$\beta _{2j}=\gamma _{20}$$

5 $$\beta _{3j}=\gamma _{30}+\gamma _{31}\cdot \text{traitSR}_{j}+\gamma _{32}\cdot \mathrm{joy}.\text{pmean}_{j}+\upsilon _{3j}$$

6 $$\beta _{4j}=\gamma _{40}+\gamma _{41}\cdot \text{traitSR}_{j}+\gamma _{42}\cdot \mathrm{joy}.\text{pmean}_{j}+\upsilon _{4j}$$

7 $$\beta _{5j}=\gamma _{50}$$

8 $$\beta _{6j}=\gamma _{60}$$

9 $$\beta _{7j}=\gamma _{70}$$

Random effects were estimated for the linear and quadratic effects of task difficulty. All models were estimated using the ‘nlme’ package in the R environment (Pinheiro et al. 2020; R Core Team 2020). Pseudo-*R*^2^ measures (R. Xu 2003) were computed as effect size estimates. A conventional α‑level of 0.05 (two-tailed) was assumed for all analyses.

## Results

### Descriptive statistics

Table 1 presents the descriptive statistics of the sample and the correlations of the variables on the between-person level.

**Table 1:** Descriptive statistics (mean ± SD or *n* (%)), ranges, and correlations of the sample of students

Variable	*M* (*SD*) or *n* (%)	Observed range [Possible range]	1	2	3	4	5	6	7	8
*1. Gender*^*a*^										
Male	276 (51.6)	–	1	–	–	–	–	–	–	–
Female	256 (47.9)									
*2. Age*	9.69 (2.80)	6–19	0.04	1	–	–	–	–	–	–
*3. School type attended*^*b*^										
Academic track of secondary school	127 (23.7)	–	−0.05	−0.61*	1	–	–	–	–	–
Other school type	408 (76.3)									
*4. Parental education*^*c*^										
University degree	330 (61.7)	–	0.05	0.06	0.07	1	–	–	–	–
Other	205 (38.3)									
*5. Trait self-regulation*	5.81 (2.77)	0–10 [0–10]	0.24*	0.22*	−0.21*	−0.08	1	–	–	–
*6. Daily learning independence*	4.61 (1.50)	1–7 [1–7]	0.14*	0.14*	−0.14*	0.02	0.43*	1	–	–
*7. Task difficulty*	−0.08 (0.85)	−3–3 [−3–3]	0.00	0.23*	−0.14*	−0.03	−0.12*	−0.49*	1	–
*8. Task volume*	0.42 (0.91)	−3–3 [−3–3]	−0.05	0.06	0.03	0.02	−0.19*	−0.45*	0.51*	1
*9. Task enjoyment*	3.81 (1.33)	1–7 [1–7]	0.15*	−0.13*	0.08	−0.06	0.22*	0.36*	−0.27*	−0.40*

age is given in years

^a^ 0 = male, 1 = female

^b^ 0 = academic track of secondary school, 1 = other school type

^c^ 0 = university degree, 1 = other

*p < 0.05

### Trait self-regulation and daily learning independence

**Fig. 1:**
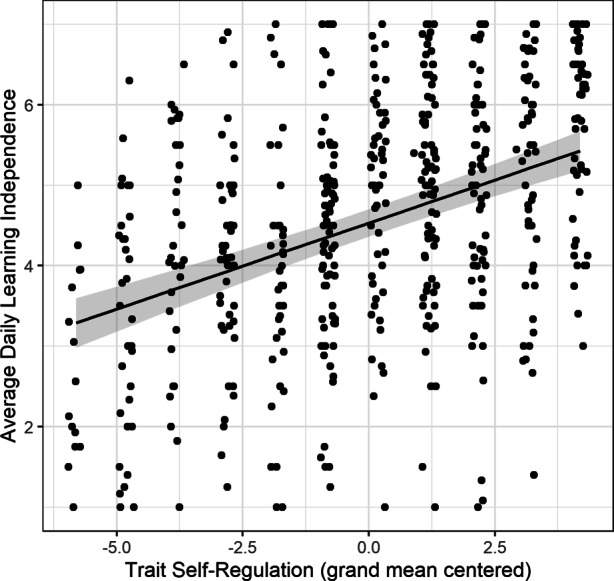
Scatterplot illustrating the association between individual students’ trait self-regulation and daily learning independence adjusted for students’ age, gender, school type attended, and parental education (each point represents one student; data points were jittered to facilitate the display)

To examine the association between *trait self-regulation* and *daily learning independence, *a multilevel model with trait self-regulation as the predictor (centred on the grand mean) and daily learning independence as the dependent variable was estimated. We further controlled for students’ gender, age, and school type attended, as well as the highest parental degree obtained. Results revealed a statistically significant positive association between students’ trait self-regulation and daily learning independence, *b* = 0.214, *p* < 0.001, suggesting that students with higher trait self-regulation worked more independently. Including trait self-regulation explained 19.1% of the between-person variance in daily learning independence above and beyond the covariates. Fig. 1 depicts this association.

### Perceived task difficulty and daily learning independence

**Fig. 2:**
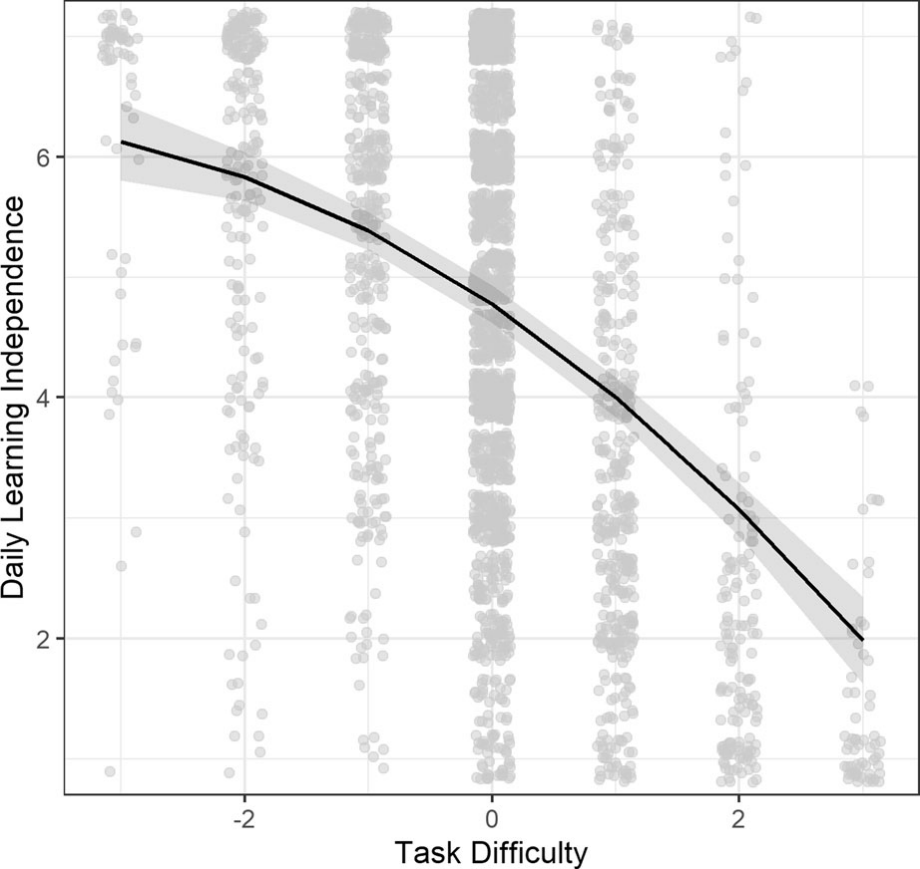
The figure depicts the predicted association between task difficulty and daily learning independence (each point represents one daily data point; data points were jittered to facilitate the display)

Results (see Table 2, Model 1) revealed the hypothesised negative quadratic association between *perceived task difficulty* and *daily learning independence, b* = −0.080, *p* < 0.001. The association between perceived task difficulty and learning independence implied in Model 1 is depicted in Fig. 2. This pattern suggests learning independence to be higher on days when tasks were perceived to be easier, while tasks perceived to be too difficult showed an increasingly negative association with daily learning independence. In addition, the results indicated a negative linear effect of task difficulty, *b* = −0.690, *p* < 0.001, indicating that the value of task difficulty for which students’ learning independence is at its maximum, is shifted to the left, that is, to easier tasks. Moreover, the findings also revealed a similar association between task volume and daily learning independence, with both a negative quadratic, *b* = −0.045, *p* = 0.001, and a negative linear effect, *b* = −0.236, *p* < 0.001. The linear and quadratic effect of task difficulty accounted for 21.6% of the within-person variance and 4.7% of the between-person variance in daily learning independence.

**Table 2:** Multilevel models predicting daily learning independence

	Model 1	Model 2	Model 3	Model 4
*Fixed Effects*				
Intercept	4.77* (0.138)	4.74* (0.132)	4.71* (0.136)	4.69* (0.131)
Gender^a^	0.325* (0.098)	0.144 (0.096)	0.231* (0.097)	0.083 (0.096)
Age	0.106* (0.024)	0.085* (0.018)	0.113* (0.024)	0.089* (0.023)
School type attended^b^	−0.140 (0.150)	−0.057 (0.144)	−0.148 (0.148)	−0.076 (0.143)
Parental education^c^	0.046 (0.101)	0.124 (0.096)	0.075 (0.099)	0.146 (0.095)
Task volume (linear)	−0.236* (0.032)	−0.226* (0.032)	−0.159* (0.032)	−0.155* (0.031)
Task volume (quadratic)	−0.045* (0.014)	−0.042* (0.014)	−0.023 (0.014)	−0.022 (0.014)
Task difficulty (linear)	−0.690* (0.032)	−0.683* (0.033)	−0.649* (0.034)	−0.642* (0.034)
Task difficulty (quadratic)	−0.080* (0.016)	−0.082* (0.016)	−0.079* (0.017)	−0.082* (0.017)
Trait self-regulation	–	0.155* (0.021)	–	0.142* (0.021)
Trait self-regulation × Task difficulty (linear)	–	−0.013 (0.011)	–	−0.015 (0.011)
Trait self-regulation × Task difficulty (quadratic)	–	−0.012* (0.006)	–	−0.009 (0.005)
Task enjoyment (WP)	–	–	0.284* (0.023)	0.284* (0.023)
Task enjoyment (WP) × Task difficulty (linear)	–	–	−0.026 (0.017)	−0.025 (0.017)
Task enjoyment (WP) × Task difficulty (quadratic)	–	–	−0.018* (0.009)	−0.017 (0.009)
Task enjoyment (BP)	–	–	0.280* (0.045)	0.217* (0.045)
Task enjoyment (BP) × Task difficulty (linear)	–	–	−0.029 (0.023)	−0.017 (0.023)
Task enjoyment (BP) × Task difficulty (quadratic)	–	–	−0.005 (0.011)	−0.001 (0.011)
*Random Effects (Standard Deviations)*				
Intercept	1.037	0.974	1.026	0.974
Task difficulty (linear)	0.244	0.229	0.235	0.217
Task difficulty (quadratic)	0.106	0.096	0.098	0.098
Level 1 Residual	1.183	1.185	1.134	1.134

The table depicts unstandardized effects and standard errors for fixed effects (in parentheses) and random effects (standard deviations) of the multilevel models predicting daily learning independence

Number of observations = 2682 (Models 3 + 4) − 2731 (Models 1 + 2)

Number of participants = 517 (Models 3 + 4) − 523 (Model 1 + 2)

*WP* = within-person, *BP* = between-person

^a^ 0 = male, 1 = female

^b^ 0 = academic track of secondary school, 1 = other school type

^c^ 0 = university degree, 1 = other

*p < 0.05

**Fig. 3:**
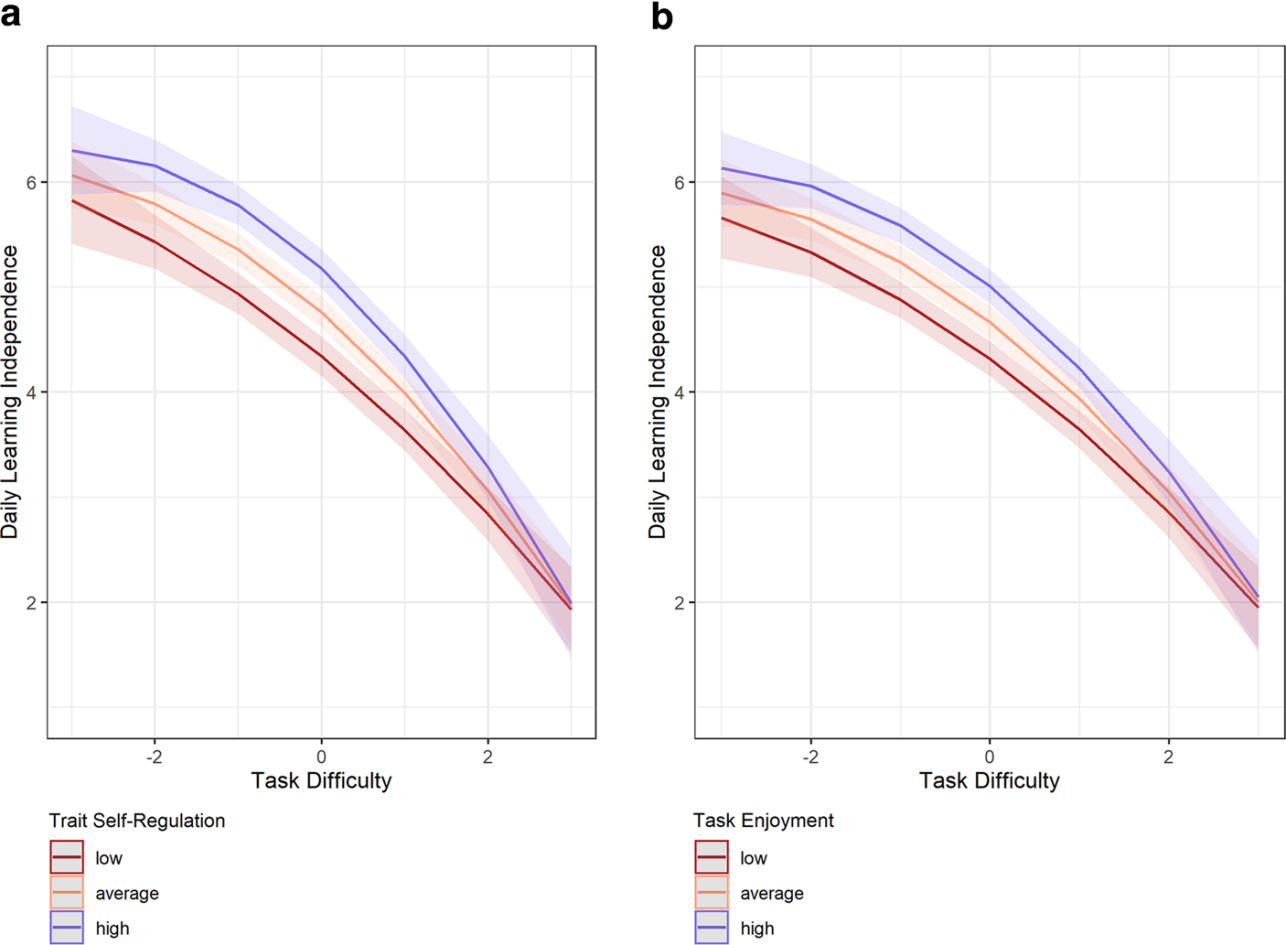
The figure depicts the predicted association between task difficulty and daily learning independence separated by values on trait self-regulation (**a**) and separated by values on task enjoyment (**b**; person-mean centred) (levels of the moderators were split at trait self-regulations’ grand mean and task enjoyments’ person mean ±1 *SD*)

We next examined whether the association between perceived *task difficulty* and *daily learning independence *was moderated by *trait self-regulation*. Results (see Table 2, Model 2) showed a statistically significant interaction between trait self-regulation and the quadratic effect of task difficulty, *b* = −0.012, *p* = 0.035. Notably, the effect was in the non-anticipated direction, with a more pronounced quadratic effect for students with higher trait self-regulation. Fig. 3a shows the model-implied quadratic association between task difficulty and daily learning independence separately for students with low, average, and high trait self-regulation (high and low trait self-regulation were operationalised as one standard deviation above and below the sample average). This figure illustrates that trait self-regulation was most strongly associated with daily learning independence at average levels of task difficulty: when the task was too difficult, the effects of trait self-regulation on daily learning independence were attenuated and virtually eliminated. Comparing the random slope variance to the corresponding estimate in a model without the cross-level interaction showed that trait self-regulation accounted for 2.5% of the between-person differences in the quadratic effect (the curvature) of task difficulty.

Model 3 revealed a statistically significant main effect of *task enjoyment* on *daily learning independence *on both the within-person and the between-person level. Students with, according to their parents, higher average task enjoyment, were also higher in their average learning independence across the study, *b* = 0.280, *p* < 0.001. Similarly, on days with higher task enjoyment, they also showed higher than average learning independence as reported by their parents, *b* = 0.284, *p* < 0.001. We predicted that state task enjoyment would moderate the quadratic effect of task difficulty on daily learning independence. While the respective interaction effect was statistically significant, *b* = −0.018, *p* = 0.038, it was not in the anticipated, but the opposite direction. Fig. 3b shows that, similar to the findings for trait self-regulation, the quadratic effect of task difficulty was less pronounced on days when students reported less task enjoyment. Notably, the interaction of daily task enjoyment with task difficulty accounted for only 0.3% of the within-person variance in daily learning independence. Finally, when both trait self-regulation and task enjoyment were added simultaneously (Model 4), the interaction effect of quadratic task difficulty with (time-varying) task enjoyment was no longer statistically significant, *b* = −0.017, *p* = 0.057. The respective interaction effect with trait self-regulation was no longer statistically significant either,* b* = −0.009, *p* = 0.110.

## Discussion

The present study aimed to investigate (a) whether students’ *trait self-regulation* was associated with their *daily learning independence *during homeschooling. Additionally, it aimed to (b) examine the effect of the perceived *task difficulty* of the learning tasks on students’ *daily learning independence*. It further aimed to examine whether (c) students’ *trait self-regulation* moderated this association. Finally, (d) the role of *task enjoyment* as a moderator of the relation between perceived *task difficulty* and *daily learning independence *was examined.

### Association of trait self-regulation and daily learning independence

The results supported the expectation of a positive association between students’ *trait self-regulation* and their *daily learning independence*. In particular, students with higher trait self-regulation learned more independently, which may be seen as an indicator of higher daily state self-regulation. As students with higher trait self-regulation should be expected to be better able to judge when they require help to overcome daily learning difficulties, while additionally being more actively engaged with the learning task and trying to solve it on their own before demanding assistance (Pintrich 2000; Duckworth et al. 2014; Zimmerman 1990), they may be assumed to ask for support less frequently than students with lower trait self-regulation. When demanding support, they should furthermore be better able to point out their precise needs, which should allow helpers to assist more effectively, thus further limiting the need for repeated requests (Karabenick and Gonida 2018; Newman 2008). In contrast, students with lower trait self-regulation can be expected to request support even though they have hardly engaged with the learning task yet (e.g., Zentall 2005). Thus, they might have difficulties identifying and expressing their precise needs, whereby helpers can only assist less effectively. This could result in increased total support time, and hence decreased learning independence (DuPaul and Stoner 2014; Grolnick and Slowiaczek 1994; Marchand and Skinner 2007; J. Xu and Corno 1998). Daily learning independence might therefore be considered an approximation for students’ state self-regulation and the present study’s results suggest that students’ trait self-regulation is associated with their state self-regulation during learning.

### Association of task difficulty and daily learning independence

The results furthermore lent support to the hypothesis that perceived *task difficulty* should be negatively associated with students’ *daily learning independence*. Students worked more independently when tasks were found to be easier, while independence in completing tasks lowered progressively with more difficult tasks. This result is in accordance with expectations derived from research demonstrating that individuals tend to disengage from tasks considered to exceed their abilities (i.e., self-regulation difficulties; Boekaerts and Corno 2005; Locke and Latham 1990). Additionally, students can be expected to increasingly seek for assistance when realising their need for support, which should increase with more difficult tasks (Karabenick and Gonida 2018; Marchand and Skinner 2007; Newman 2008). Contrasting our expectations, too easy tasks were not associated with lower daily learning independence. Consequently, students’ state self-regulation during learning should only be expected to have lowered with more difficult tasks.

Further contrasting our prediction that lower *trait self-regulation* would be associated with a stronger negative effect of *task* *difficulty* on *daily learning independence*, our results suggested that this association was in fact more pronounced in students with higher trait self-regulation. When facing more difficult tasks, parents reported students with higher trait self-regulation to show stronger reductions in their daily learning independence as compared to students with lower trait self-regulation. In terms of students’ state self-regulation, this result might indicate that students with higher trait self-regulation experienced a stronger reduction in their state self-regulation when working on more difficult tasks. However, this finding might also be explained by the idea that students who usually tend to work relatively independently (i.e., those with higher trait self-regulation) may experience a greater decline in daily learning independence compared to students who generally tend to require a lot of assistance (i.e., those with lower trait self-regulation), simply because there is greater leeway. A complementary interpretation of this interaction pattern would be that differences in children’s trait self-regulation become less relevant in situations of very easy or very difficult tasks, and in these situations task characteristics might outweigh between-person characteristics in determining the extent to which children require assistance for working on school related tasks. Consequently, the result that students with higher trait self-regulation seem to experience a stronger reduction in their state self-regulation when working on more difficult tasks should be interpreted with caution as methodological constraints might apply.

### Association of task enjoyment and daily learning independence

Finally, both within and between students, higher *task enjoyment* was associated with higher *daily learning independence*. This is, students generally worked more independently when enjoying the tasks more, while individual students also worked more independently on days they enjoyed the tasks more. With regard to students’ self-regulation, these results might indicate that, both on a daily basis and in general, more enjoyable tasks should support students’ self-regulation more. This finding is in accordance with empirical evidence suggesting that higher task enjoyment should be positively associated with students’ daily self-regulation during learning (Judge et al. 2005; Koestner et al. 2008). In particular, task enjoyment should support the adoption of externally determined goals (i.e., ‘have-to’ goals) as individually pursued goals (i.e., ‘want-to goals’), whereby momentary self-regulation is improved (Milyavskaya et al. 2015; Muraven et al. 2008; Sieber et al. 2019).

However, our expectation that higher task enjoyment would reduce the negative impact of increasing task difficulty on students’ daily learning independence was not supported. On the contrary, higher task enjoyment coincided with a stronger negative association between task difficulty and daily learning independence and thus presumably also students’ state self-regulation. This finding might, however, be explained by the idea that enjoyable tasks encouraged students to be more persistent in solving even difficult tasks, whereby they increasingly requested support (Duckworth et al. 2007; Duckworth and Quinn 2009; Wolters and Hussain 2015). Nevertheless, we hasten to add that this not anticipated interaction effect explained less than 1% of the day-to-day variance in students’ learning independence, suggesting that task enjoyment only plays a minor role in modulating the effect of task difficulty on learning independence.

### Practical implications

The present findings have important practical implications. First, the finding that students’ daily learning independence as an indicator of daily self-regulation was lower with more difficult tasks, but did not lower with easy tasks, might be viewed to indicate that teachers should carefully consider task difficulty when designing learning tasks for homeschooling purposes. As illustrated in Fig. 3, the interaction pattern additionally suggested that both too easy and too difficult tasks eliminated the effect of trait self-regulation on daily learning independence. That is, our findings suggest that task difficulty might override pre-existing differences in trait self-regulation when it comes to predicting daily self-regulation. This finding emphasises the crucial role of task attributes for students’ daily self-regulation during homeschooling. In particular, the results indicate that teachers should certainly consider that students ask their parents for support more frequently when daily learning materials are more difficult. This could imply that children whose parents are less reliable or less available to support in completing daily school work (e.g., due to their own work) could be disadvantaged. Additionally, this could be relevant when parents cannot provide support because they have difficulties understanding the learning material themselves. Nevertheless, task difficulty cannot be lowered at will and certain difficulty levels need to be established for learners to gain knowledge (i.e., zone of proximal development; Meece and Daniels 2008; Vygotsky 1978). Additionally, working on more difficult tasks might be beneficial beyond students’ learning as it could be expected to be effective as a self-regulation training (Koole et al. 2012). In sum, these considerations therefore certainly underscore the urgent need for teachers to be available as contact persons (e.g., via telephone or video chat) for children to assist in working on the daily learning tasks during homeschooling periods.

Additionally, as the results demonstrated that students’ daily task enjoyment was positively related to their daily learning independence and thus presumably to their state self-regulation, teachers should consider to what extent the learning tasks provided are enjoyable for their students during homeschooling. As higher state self-regulation may be expected to support students’ daily learning, more enjoyable tasks should lead to better learning outcomes, as has been demonstrated by a plethora of previous research (Pekrun 2006; Pekrun et al. 2002, 2017; Putwain et al. 2018). Teachers should therefore aim to provide their students with rather enjoyable tasks.

### Limitations and future research

To our knowledge, the present study was the first to examine the relevance of students’ trait self-regulation for their daily independence in working on learning materials during homeschooling in the course of the SARS-CoV‑2 pandemic, thereby additionally considering the role of further task attributes. Future studies aiming to replicate and extend the present study’s findings should consider its following limitations. First, the present study did not obtain information on students’ learning outcomes. Thus, it could not determine whether students’ independence during learning, task difficulty, and task enjoyment were at all related to students’ learning success, and hence denote variables to be considered with regard to students’ learning outcomes. Future studies should therefore aim to obtain information allowing for conclusions on students’ daily learning success to be drawn. Longitudinal information on educational attainment after return to regular school lessons could be one way to attain this information.

Second, assessing students’ daily learning independence as an indicator of students’ daily state self-regulation in the learning situation should be considered a relatively global index. While the results indicate that students’ trait self-regulation is associated with their daily learning independence, which is in line with what was hypothesised, the results should also be understood as to encourage future research to take further steps to more precisely illustrate relations between students’ trait self-regulation and daily learning behaviour. Thus, much more detailed conclusions will be licensed.

Third, data in the present study were obtained through parents only. However, it cannot be ruled out that, particularly for older children who will generally work on learning tasks more independently, more reliable and valid data reflecting students’ actual estimations regarding task difficulty and task enjoyment could be obtained by interviewing students themselves. Future studies should therefore consider to assess relevant variables through interviews with children instead or in addition to parents. Additionally, since all information were obtained from one source only, perceiver effects and common method influences need to be considered as potential biases of the findings reported here.

Fourth, the majority of students reported about in the present investigation attended primary school. The results presented here are therefore primarily based on information on younger students. Whether or not the findings would generalise to a sample of primarily older students or to students preparing for their school leaving examinations therefore remains unknown.

Finally, parents were recruited via announcements posted to social media platforms, contacts to schools and parent-teacher associations, as well as a press release issued by the authors’ institution, which was further distributed through German newspapers. To participate in the study, parents had to indicate their interest. Thus, the recruited sample was based on self-selection. Parental background (education and household income) suggested that the sample was positively selected. To account for differences in socioeconomic status (SES) between families, parental education (i.e., university degree vs. other) was controlled for (note that this covers just one—yet in the present context arguably the most relevant—facet of SES). The generalisation of the results obtained to the general population is therefore not warranted.

## Conclusion

The present work examined the role of both students’ trait self-regulation as a person characteristic and task difficulty and task enjoyment as features of the learning tasks students worked on during the SARS-CoV-2-induced homeschooling period in Germany for students’ daily self-regulation. The results supported the hypothesis of an association between students’ trait self-regulation and their state self-regulation operationalised as their daily learning independence. Additionally, the results supported the important role of task features such as task difficulty and task enjoyment for students’ daily self-regulation. Thus, the present investigation encourages the consideration of both person characteristics and features of the learning tasks students work on during homeschooling when designing learning tasks to support students’ self-regulation.

## Footnotes

1 Note that the wording of this item was changed in the course of the study. The wording was changed about one week after the beginning of the study’s daily diary part based on open responses provided by the parents. These responses indicated that children often received tasks they were supposed to work on over several days, rather than daily task packages to work on a particular day. The wording was changed from ‘Did your child receive learning materials or tasks from school today?’ to ‘Has your child worked on materials or tasks for school today? This either refers to tasks designed for this particular day, or tasks that can be worked on over several days.’.
